# Qualitative and quantitative studies on two commercial specifications of *Polygonatum odoratum*

**DOI:** 10.3389/fchem.2023.1146153

**Published:** 2023-02-23

**Authors:** Yi Nan, Haizhen Liang, Xu Pang, Wei Zheng, Yuhao Shi, Xiaojuan Chen, Jie Zhang, Juan Song, Baiping Ma

**Affiliations:** ^1^ Beijing Institute of Radiation Medicine, Beijing, China; ^2^ Graduate School, Tianjin University of Traditional Chinese Medicine, Tianjin, China

**Keywords:** *Polygonatum odoratum*, UHPLC-Q-TOF/MS, UHPLC-CAD, commercial specification, quality control

## Abstract

The rhizoma of *Polygonatum odoratum* (PO) is used to treat yin injuries of the lung and stomach in traditional Chinese medicine. The chemical constituents of this herb are steroidal saponins, homoisoflavanones, and alkaloids. Xiangyuzhu (XPO) and Guanyuzhu (GPO) are available in the market as two specifications of the commodity. Nonetheless, systematic research on the identification and comparison of chemical constituents of these two commercial specifications is yet lacking. Herein, an integrated method combing ultra-high-performance liquid chromatography-quadruple time-of-flight mass spectrometry (UHPLC-Q-TOF/MS) with ultra-high-performance liquid chromatography-charged aerosol detection (UHPLC-CAD) was employed for the comprehensively qualitative and quantitative analyses of PO. A total of 62 compounds were identified by UHPLC-Q-TOF/MS, among which 13 potential chemical markers were screened out to distinguish two commercial specifications. Subsequently, the absolute determination method for polygodoraside G, polygonatumoside F, and timosaponin H1 was established and validated by UHPLC-CAD. The contents of the three compounds were 13.33–236.24 μg/g, 50.55–545.04 μg/g, and 13.34–407.83 μg/g, respectively. Furthermore, the ratio of timosaponin H1/polygodoraside G could be applied to differentiate the two specifications. Samples with a ratio <2 are considered XPO and >5 are considered GPO. Therefore, the above results provide a valuable means for the quality control of PO.

## 1 Introduction

Plants of genus *Polygonatum* have been extensively used as traditional Chinese medicine. The Chinese Pharmacopoeia records the rhizome of *Polygonatum odoratum* (PO) as a yin-nourishing medicine “Yuzhu”, which can be used for the treatment of diabetes and cough (Zhao et al., 2020; Li et al., 2021). PO is widely distributed in the temperate regions of Eurasia (Xia et al., 2022), growing in the forest or on the shady slopes of mountains (Feng et al., 2022). The major chemical constituents of PO are steroidal saponins, homoisoflavanones, alkaloids, and polysaccharides (Zhao et al., 2018; Zhao et al., 2019). In recent years, PO has gained increasing attention (Ning et al., 2018; Wang H J et al., 2018; Pang et al., 2021; Zhou et al., 2021), but there is no systematic study on the chemical identification of PO. Xiangyuzhu (XPO) and Guanyuzhu (GPO) are available in the market as two main commercial specifications with different origins and prices. In the book “S*tandard compilation of authentic medicinal materials*,” the appearance of XPO is thick, long, translucent, and light yellow in color, and that from Hunan is considered an authentic medicinal material, which occupies the majority of the Chinese market. GPO is a wild product in Northeast China, Inner Mongolia, and Hebei, and its cost is lower than XPO. Nonetheless, XPO is more frequently used as the raw material of dietary supplements, while GPO is more inclined to medicinal use. Currently, the quality control of PO was the only focus on the polysaccharide content (Jing et al., 2022), whereas some studies have pointed out that the polysaccharide content of GPO is higher than that of XPO (Tian et al., 2014). However, no comparison has been made between the other small molecule chemicals of these two commercial specifications to date. Therefore, it is essential to develop a specific method to distinguish the two commercial specifications in terms of chemical ingredients.

Ultra-high-performance liquid chromatography-quadrupole time-of-flight mass spectrometry (UHPLC-Q-TOF/MS) is a capital analytical tool with good resolution, excellent sensitivity, and strong structural characterization capability and has also been used in the qualitative analysis of *Polygonatum* genus (Huang et al., 2020; Sui et al., 2022). Off-line solid phase extraction (SPE) has been used in the pretreatment of complex samples to remove polysaccharides that cannot be retained on C18 columns and enrich targeted components for analysis (Li et al., 2019). The present study aimed to establish a qualitative method by coupling off-line SPE with UHPLC-Q-TOF/MS to characterize PO in positive and negative ion modes. The measured molecular weight plus the fragment ion information obtained by MS/MS could identify the structures of the chemical components (Zhao et al., 2013). Multivariate statistical analyses [for example, principal component analysis (PCA), orthogonal partial least squares discriminant analysis (OPLS-DA), and variable importance in the projection (VIP) plot] were used to identify the potential differential components among XPO and GPO (Pan et al., 2021).

As an aerosol-based universal detector, charged aerosol detector (CAD) can be characterized by high sensitivity, broad dynamic range, less interanalyte response variability, and improved reproducibility, which has gained increasing popularity for LC analysis of organic compounds with poor UV chromophores (Haidar Ahmad et al., 2019; Zheng et al., 2022). The fitting method of regression equation relies on the concentration range. In many cases, the linearity of CAD response is sufficient over the range of interest (Pawellek et al., 2021). Due to its high sensitivity, the limit of detection (LOD) and limit of quantification (LOQ) may be at the nanogram level (Zhang et al., 2019). Saponins are a class of active components used as key quality control indicators for content determination in many studies (Zhang et al., 2019; Nan et al., 2021). The furostonal saponin polygonatumoside F showed the higher content in the PO samples and may be served as the chemical marker for quality control (Liu et al., 2018). In the present study, the other two components polygodoraside G and timosaponin H1 were screened out as major chemical markers in XPO and GPO. In order to assess the quality of PO, UHPLC-CAD method was used, and a content ratio of timosaponin H1/polygodoraside G was proposed to differentiate the two commercial specifications of PO.

## 2 Materials and methods

### 2.1 Materials and reagents

Acetonitrile (HPLC grade) was purchased from Fisher Scientific Co. (Loughborough, United Kingdom). Distilled water was purchased from Watsons. Formic acid (HPLC grade) was purchased from Acros Co. Ltd. (NJ, United States). The other reagents of analytical grade were also obtained commercially (Beijing, China).

A total of 27 batches of representative PO were collected or purchased from Hunan (XPO, S1-S14) and Heilongjiang (GPO, S15-S27) provinces of China. The identity of all samples was confirmed by Prof. Bao-lin Guo of the Institute of Medicinal Plant Development, Chinese Academy of Medical Science&Peking Union. A total of 22 reference standards (purity >95%), polygodoraside A-H, typaspidoside H, typaspidoside L, polygonatumoside F, timosaponin H1, (25*S*)-26-*O*-(*β*-D-glucopyranosyl)-furost-5-en3*β*,22*α*,26-triol 3-*O-β*-D-glucopyranosyl-(1→2)-*β*-D-glucopyranosyl-(1→4)-*β*-D-glucopyranoside, officinalisnin II, 25-*epi*-officinalisnin II, (3*R*)-5,7-dihydroxy-6,8-dimethyl-3-(4′-hydroxybenzyl)-chroman-4-one, (3*R*)-5,7-dihydroxy-6-methyl-8-methoxy-3-(4′-hydroxybenzyl)-chroman-4-one, (3*R*)-5,7-dihydroxy-6-methyl-3-(4′-hydroxybenzyl)-chroman-4-one, *N-trans-p*-coumaroyloctopamine, *N-trans*-feruloyloctopamine, *(E)*-3-(4-hydroxy-3-methoxybenzylidene)-4-(4-hydroxyphenyl)-pyrrolidin-2-one, and 3-(4-hydroxy-3-methoxy-phenyl)-acrylic acid carboxymethyl ester, were isolated in our laboratory and their structures were confirmed by comparing their MS and NMR spectral data with those described previously (Pang et al., 2020).

### 2.2 Preparation of samples and standard solutions

An equivalent of 1.0 g accurately weighed fine powder (<40 mesh) of each sample was mixed with 20 mL of 70% ethanol was added. After ultrasonication for 60 min, the solutions were cooled to room temperature, and the weight loss was replaced with 70% aqueous EtOH. Then, the solutions were filtered through a 0.45 µm membrane before quantitative analysis. A volume of 10 mL of the supernatant was concentrated under reduced pressure and diluted to 4 mL with deionized water. A solid-phase extraction cartridge (C18-SPE, 6 mL) was activated with 10 mL of methanol, rinsed with 10 mL of water, and then reconstituted in 4 mL solution to load the sample; first, 10 mL water was used for elution, followed by 4 mL of 95% ethanol. Finally, the ethanol eluate was collected and filtered through a 0.22-μm membrane for qualitative analysis.

The stock solutions of 22 standard references were prepared in acetonitrile at a final concentration of 0.1 mg/mL and analyzed by UHPLC-Q-TOF/MS. All the solutions were stored at 4°C for further study.

### 2.3 Qualitative analysis by UHPLC-Q-TOF/MS

UHPLC-Q-TOF/MS analysis was performed on an ACQUITY UHPLC™ system (Waters Corp. Milford, MA, United States) coupled with a Synapt G1 MS system (Waters Corp. Manchester, United Kingdom). A Waters ACQUITY UHPLC HSS T3 column (100 × 2.1 mm, 1.8 μm) was used for the analysis with the column temperature at 40°C. The mobile phases were water with 0.1% formic acid (A) and acetonitrile (B). The gradient used was as follows: 0–2 min, 5%→15% B; 2–18 min, 15%→37% B; 18–25 min, 37%→50% B; 25–27 min, 50% B; 27–28 min, 50%→5% B and 28–30 min, 5% B. The flow rate was 0.5 mL/min. The injection volume of the sample was 5 μL.

The data acquisition mode was MS^E^. The data were obtained at 50–1500 Da. The source temperature was 100°C, the desolvation temperature was 450 °C with desolvation gas flow 850 L/h, leucine enkephaline was used as lock mass, and the capillary voltage was 3 kV. At low CE scan, the cone voltage was 30 V for ESI, and the collision energy was 6 eV (trap) and 4 eV (transfer), while it was 40–60 eV ramp (trap) and 12 eV (transfer) for ESI^−^ and 15–25 eV ramp (trap) and 12 eV (transfer) for ESI^+^. The instrument was controlled by MassLynx 4.1 software (Waters Corp.).

### 2.4 Quantitative analysis by UHPLC-CAD analysis

UHPLC-CAD analysis was performed on the Thermo Vanquish UHPLC system (ThermoFisher Scientific, Germering, Bavaria, Germany). A Waters ACQUITY™ UHPLC HSS T3 column (100 × 2.1 mm, 1.8 µm) was used at a column temperature at 40°C, and the sample temperature was 10°C. The mobile phases were water with 0.1% formic acid (A) and acetonitrile (B). The gradient was as follows: 0–3 min, 10%→20% B; 3–12 min, 20% B; 12–13 min, 20%→22% B; 13–24 min, 22% B; 24–25 min, 22%→95% B; 25–27 min, 95% B; 27–28 min, 95%→10% B; 28–30 min, 10% B. The injection volume of the sample was 5 µL. The data collection was 5, and the filtration was for 3.6 s.

### 2.5 Validation of UHPLC-CAD analysis

The linearity of regression curves was tested by diluting the mixed stock solution to a series of concentrations of working solutions, and each was subjected to UHPLC analysis. The lower LODs and LOQs were determined by analyzing the serially diluted reference solutions of each compound until the signal-to-noise (S/N) proportion was about 3 and 10, respectively. The samples were analyzed in six replicates. The stability was tested by analyzing the sample solutions at different time points (0, 2, 4, 6, 8, 12, and 24 h). The RSD values of peak areas were calculated to examine the precision, repeatability, and stability of the quantitative method. Its accuracy was evaluated by recovery experiments. A specific amount of individual reference standards was spiked into PO sample. Five fortified samples were extracted and analyzed as described above. The recovery value (%) was calculated by the following equation: recovery (%) = 100 × (detected amount − original amount)/spiked amount.

### 2.6 Multivariate analysis

The ESI-MS^E^ centroid data were processed by MarkerLynx version 4.1 (Waters Corp. Manchester, United Kingdom). The analysis included deconvolution, alignment, and data reduction to obtain a list of mass and retention time pairs with the corresponding areas for all the detected peaks from each file in the dataset. The processed data list was then imported by the PCA and OPLS-DA. All the test groups were discriminated in the PCA to investigate whether different groups could be separated. The method parameters were as follows: retention time range, 1–25 min; mass range, 100–1500 Da; mass tolerance, 0.02 Da; 6.00 for noise elimination level, 5% of the base peak intensity of minimum intensity; 0.20 min for RT tolerance. Moreover, isotopic peaks were excluded from analysis. Then, OPLS-DA was carried out to discriminate the ions contributing to the classification of the samples. The results were visualized in a score plot to show group clusters, and a VIP plot showed variables contributing to the classification.

## 3 Results and discussion

### 3.1 Characterization of chemical compounds in PO

To obtain satisfactory separation and high analytical efficiency, a series of preliminary experiment conditions, including chromatographic column particle size, mobile phase composition, and column temperature, were optimized. Various columns, such as ACQUITY UHPLC BEH C18 (2.1 mm × 100 mm, 1.7 μm) and CORTECS T3 (2.1 mm × 100 mm, 1.6 μm) were compared. Finally, the result was obtained on UHPLC HSS T3 column (100 × 2.1 mm, 1.8 µm). The acetonitrile-water system showed better separation and a more satisfactory peak shape than the methanol-water system. Moreover, 0.1% formic acid was added to the ACN-water system to enhance the peak capacity and improve the peak shape of saponins and flavonoids. The column showed excellent separation performance at 40°C.

**FIGURE 1:**
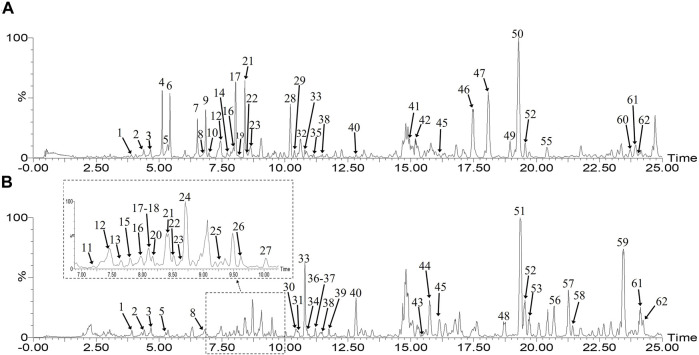
Base peak ion (BPI) chromatograms of XPO **(A)** and GPO **(B)** in the negative mode by UHPLC-Q-TOF/MS.

Simultaneously, for the identification of the chemical components in PO by UHPLC-Q-TOF/MS, the MS parameters, such as ionization mode, capillary voltage, and different collision energy ranges, were optimized. The herbal extract samples were analyzed in the positive and negative ion modes with the same LC conditions. As shown in Supplementary Table S1, 62 compounds were identified from the PO extract tentatively. The characterization was validated by the data of high-resolution mass spectrometry (Figure 1). The combination of the measured molecular weight with the fragment ion information obtained by collision-induced dissociation (CID) identified the structures and deduced that alkaloids, flavonoids, and steroidal saponins were the major constituents of the components.

#### 3.1.1 Alkaloid derivatives

Alkaloids are nitrogen-containing basic organic compounds existing in nature. A total of four pairs of alkaloid components were identified in Supplementary Table S1; their ion chromatographic signals were well-displayed in positive and negative ion modes. Each group of alkaloids had *cis* and *trans* structures, and the isomers could be determined by referring to the retention time of reference materials.

**FIGURE 2:**
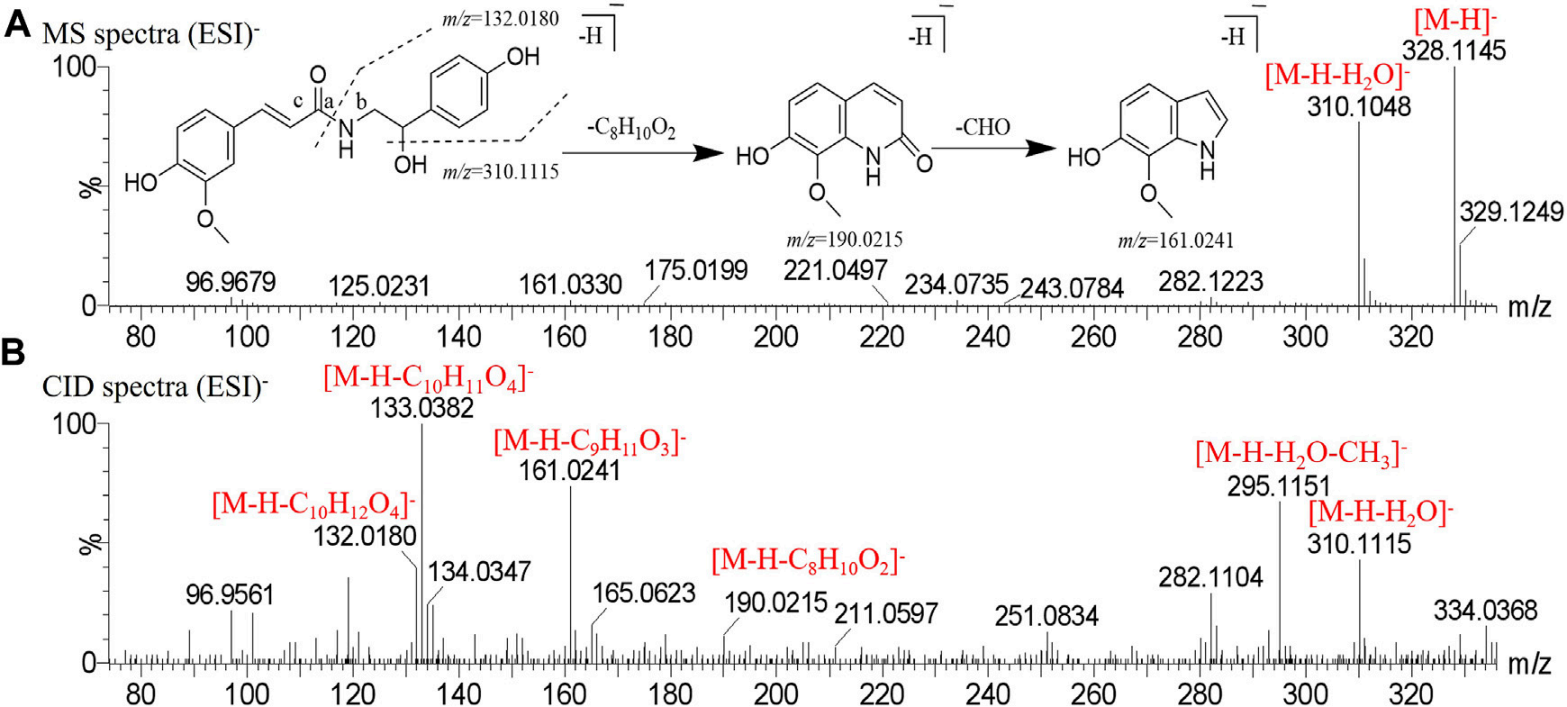
Mass spectrogram in negative mode and fragmentation pathways of *N*-*cis*-feruloyloctopamine. **(A)** MS spectra. **(B)** CID spectra.

Peak two produced a deprotonated molecular ion at *m/z* 328.1145 [M-H]^-^ and *m/z* 310.1048 [M-H-H_2_O]^-^ in the MS^1^ spectrum. In the CID spectrum, the peak produced the fragment ion at *m/z* 161.0241 [M-H-C_9_H_11_O_3_]^-^ was broken at the b bond resulting from McFarland’s rearrangement cleavage, and also obtained the fragment ion *m/z* 190.0215 [M-H-C_8_H_10_O_2_]^-^; then, a molecule of CHO was removed to obtain the fragment ion *m/z* 132.0180 [M-H-C_10_H_12_O_4_]^-^ was generated by the cleavage of the bond based on the loss of a molecule of H_2_O and McFarland’s rearrangement. Peak two was tentatively identified as *N-cis*-feruloyloctopamine. The comparison of retention time showed that the alkaloids with the *cis* structure peaked more than those with *trans* structures. Also, other alkaloids were identified by this rule, and the mass spectrogram in the negative mode and the cracking pathway are shown in Figure 2.

#### 3.1.2 Flavonoids

The homoisoflavanones of PO are critical bioactive compounds (Wang Y et al., 2018; Xia G. H. et al., 2021; Xia G. H. et al., 2021), and the potential biosynthetic pathway for isoflavonoid formation has been defined in the PO (Zhang et al., 2020). It can generate characteristic fragment ions by RAD cleavage in the C ring at high energy levels (Ying et al., 2020), and the bond connecting the two sides of the methylene group between the B and C rings is broken to form other characteristic fragment ions.

**FIGURE 3:**
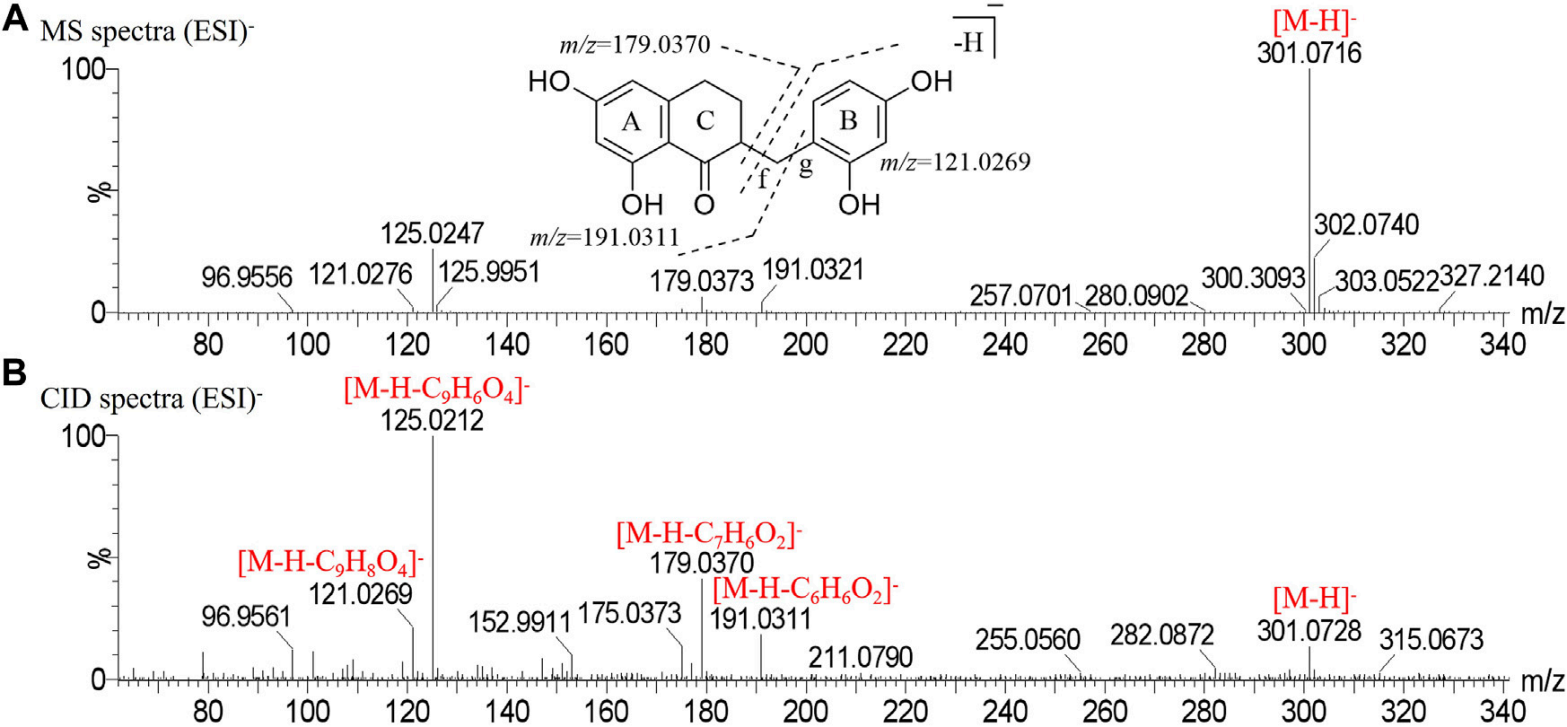
Mass spectrogram in negative mode and fragmentation pathways of disporopsin. **(A)** MS spectra. **(B)** CID spectra.

Peak 32 produced a deprotonated molecular ion at *m/z* 301.0716 [M-H]^-^ in the MS^1^ spectrum. In the CID spectrum, the peak produced characteristic fragment ions *m/z* 179.0370 [M-H-C_7_H_6_O_2_]^-^ and *m/z* 121.0269 [M-H-C_9_H_8_O_4_]^-^ due to *α*-cleavage at the f bond (Ren et al., 2021) and the characteristic fragment ion *m/z* 191.0311 [M-H-C_6_H_6_O_2_]^-^ due to *α*-cleavage at the g bond. Finally, peak 32 was determined to be disporopsin, and the mass spectrogram in the negative mode and the potential cracking pathway are shown in Figure 3.

#### 3.1.3 Steroidal saponins

Diosgenin and yamogenin are the main parent nucleus types in PO, and the C-3 position is often substituted by 3-4 sugar groups. The sugar group mainly consists of arabinose, glucose, xylose, and rhamnose. The negative ion mode displays deglycosylation fragments, while the positive ion mode presents patent ion characteristic fragments. Thus, these compounds could be identified by the combination of the positive and negative ion modes.

**FIGURE 4:**
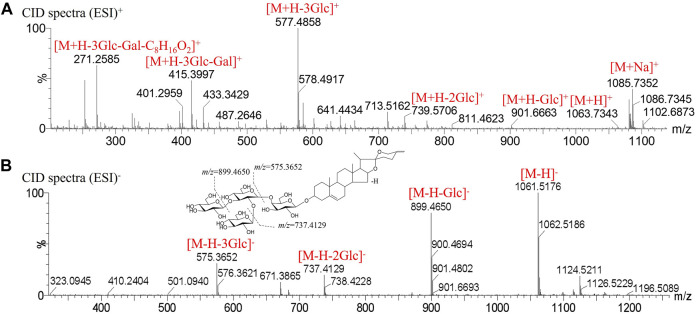
Mass spectrogram in positive, negative mode and fragmentation pathways of 3-*O-β*-D-glucopyranosyl-(1→2)-[*β*-D-glucopyranosyl-(1→3)]-*β*-D-glucopyranosyl (1→4)-*β*-D-galacopyranosyl-diosgenin. **(A)** CID spectra in positive mode. **(B)** CID spectra in negative mode.

Peak 60 produced a deprotonated molecular ion at *m/z* 1061.5176 [M-H]^-^ in the MS^1^ spectrum. In the negative CID spectrum, the peak produced the fragment ions at *m/z* 899.4650 [M-H-Glc]^-^, *m/z* 737.4129 [M-H-Glc-Glc]^-^, and *m/z* 575.3652 [M-H-Glc-Glc-Glc]^-^. In the positive CID spectrum, the peak produced the fragment ions at *m/z* 739.5706 [M + H-Glc-Glc]^+^, *m/z* 577.4858 [M + H-Glc-Glc-Glc]^+^, and *m/z* 415.3997 [M + H-Glc-Glc-Glc-Gal]^+^. The presence of the characteristic ion *m/z* 271.2585 [M + H-Glc-Glc-Glc-Gal-C_8_H_16_O_2_]^+^ means that the composition is the same as the parent nucleus of 3-*O-β*-D-glucopyranosyl-(1→2)-[*β*-D-xylopyranosyl-(1→3)]-*β*-D-glucopyranosyl (1→4)-*β*-D-galacopyranosyl-diosgenin, and the peak is finally determined to be 3-*O*-*β*-D-glucopyranosyl-(1→2)-[*β*-D-glucopyranosyl-(1→3)]-*β*-D-glucopyranosyl (1→4)-*β*-D-galacopyranosyl-diosgenin. The mass spectrogram in the positive, negative mode and possible cracking pathway are shown in Figure 4.

#### 3.1.4 Acetylated steroidal saponins

Acetylated steroidal saponins are primary metabolites that have been reported in the same genus of plants (Ahn et al., 2006). The position of the acetyl group is not fixed and could appear in the C-1 position or the sugar group inside the C-3 position. Such compounds mainly exist in GPO, and it is feasible to classify such compounds by the presence of a neutral loss of 42 Da in the mass spectrum.

For example, peak 36 produced a deprotonated molecular ion at *m/z* 1253.595 [M-H]^-^ in the MS^1^ spectrum. Typically, we observed that the precursor ion sheds 42 Da of *m/z* 1211.5792 [M-H-COCH_2_]^-^ in the CID spectrum. The peak produced the fragment ions at *m/z* 1079.5385 [M-H-COCH_2_-Xyl]^-^, *m/z* 1049.5275 [M-H-COCH_2_-Glc]^-^, *m/z* 917.4746 [M-H-COCH_2_-Glc-Xyl]^-^, and *m/z* 755.4301 [M-H-COCH_2_-2Glc-Xyl]^-^. The most fragment ion peaks are the same as timosaponin H1, which is the hydroxyacetylated precursor compound acetyl-timosaponin H1. The mass spectrogram in the negative mode of acetyl-timosaponin H1 and timosaponin H1 are shown in Supplementary Figure S1.

#### 3.1.5 Identification of novel compounds

The summary of the chromatographic rules of reference substances could be used to deduce the structure of unknown compounds to identify novel compounds. For example, the retention time of compounds with the glucose terminal group is less than that of xylose, and peak 7 can be inferred from this rule. The retention time of peak 7 was close to polygodoraside F and produced a deprotonated molecular ion at *m/z* 1255.5535 [M-H]^-^ in the MS^1^ spectrum. In the CID spectrum, the peak produced the fragment ions at *m/z* 931.4557 [M-H-Glc-Glc]^-^, *m/z* 769.3910 [M-H-Glc-Glc-Glc]^-^, and *m/z* 571.3401 [M-H-Glc-Glc-Glc-Glc-2H_2_O]^-^; these fragments were the same as those of polygodoraside F, and the retention time is relatively close. Therefore, it is inferred that there is difference between peak 7 and polygodoraside F in only the terminal sugar group; the peak 7 is finally determined to be polygodoraside F-Xyl + Glc. The mass spectrogram in the negative mode of polygodoraside F-Xyl + Glc and polygodoraside F are shown in Supplementary Figure S2.

As a result, 62 compounds were identified from PO tentatively, including 43 steroidal saponins, 11 flavonoids, 8 alkaloids, and 10 possible new components.

### 3.2 Discrimination of PO samples with two commercial specifications by PCA and OPLS-DA analysis

**FIGURE 5:**
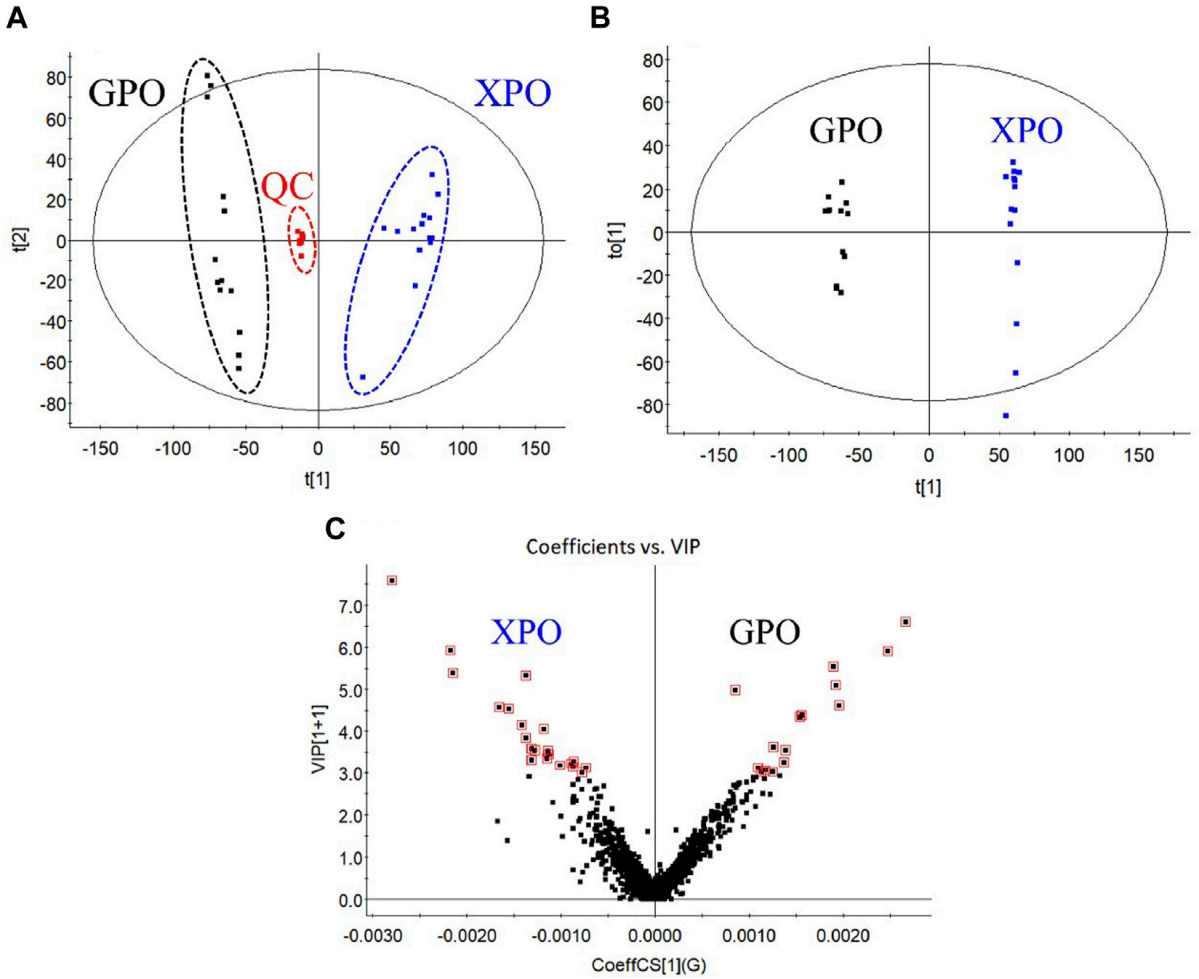
PCA score plot **(A)**, OPLS-DA score plot **(B)**, and VIP plot **(C)** of XPO and GPO.

Multivariate statistical analysis was carried out on the metabolite data to discriminate the two commercial specifications of PO. First, the obtained multivariate dataset of 27 batches of samples was analyzed by PCA. The results showed that the samples from different specifications were classified into two categories (Figure 5A), indicating a great variation in the chemical profile between XPO and GPO.

Then, the OPLS-DA model and VIP plot (Figure 5C) were established to identify the key markers that contribute to the differences between XPO and GPO, and a remarkable separation between these two specifications was also observed in the OPLS-DA score plot (Figure 5B). The model displayed 99% of the variation in the response Y (class) (R^2^Y = 99%) and also predicted 97% of the variations in the response Y (Q^2^Y = 97%). Therefore, the model could satisfactorily distinguish between the two species of samples. The VIP values (VIP >3.5) from OPLS-DA model were utilized to identify the potential differentiated variables, and 13 robust known chemical markers (including 6 flavonoids and 7 steroidal saponins) between XPO and GPO were selected and listed (Table 1). Isomer of (25*R*,22*ξ*)-hydroxylwattinoside C (peak 24) and 3*β*-hydroxy-25*S*-spiriost-3-*O*-*β*-D-glucopyranosyl (1→4)-*β*-D-galactopyranoside (peak 57) only existed in GPO samples, and their presence could be used to determine the commercial specification of PO. The differences in other components were differentiated in the content. For example, (1) compared to polygodoraside G (peak 17), no hydroxyl substitution was observed at the C-14 position of the parent nucleus of timosaponin H1 (peak 33). Peak 17 was a high content in XPO samples, while peak 33 was high in GPO samples; (2) polygonatumoside G (peak 40) was the product of deglycosylation of peak 17 with higher content in GPO samples; (3) as representative isoflavonoids components, (3*R*)-5,7-dihydroxy-6-methyl-3-(4′-hydroxybenzyl)-chroman-4-one (peak 46), (3*R*)-5,7-dihydroxy-6-methyl-8-methoxy-3-(4′-hydroxybenzyl)-chroman-4-one (peak 47), and (3*R*)-5,7-dihydroxy-6,8-dimethyl-3-(4′-hydroxybenzyl)-chroman-4-one (peak 50) showed high contents in XPO samples. These chemical markers make it possible to distinguish the two groups of PO samples; then, two conspicuously characteristic markers, polygodoraside G and timosaponin H1, were selected for further quantitative analysis to evaluate the quality of PO from two specifications.

**TABLE 1:** Selected markers responsible for discriminating two commercial specifications of PO.

Peak No.	*t*_R_ (min)	[M-H]^-^	Identification	Main existing groups
17	8.03	1257.5757	Polygodoraside G	XPO
24	8.71	933.4652	Isomer of (25*R*, 22 ξ)–hydroxylwattinoside C	GPO
28	10.21	741.4414	(22*S*)-cholest-5-ene-1*β*,3*β*,16*β*,22-tetrol-1-*O*-*α*-*L*-rhamnopyranosyl-16-*O*-*β*-D-glucopyranoside	XPO
33	10.79	1211.5667	Timosaponin H1	GPO
40	12.82	609.3631	Polygonatumoside G	GPO
42	15.19	1061.5146	Isomer of 3-*O*- β -D-glucopyranosyl-(1→2)-[*β*-D-glucopyranosyl-(1→3)]-*β*-D-glucopyranosyl (1→4)-*β-*D-galacopyranosyl-diosgenin	XPO
46	17.47	299.0952	(3*R*)-5,7-dihydroxy-6-methyl-3-(4′-hydroxybenzyl)-chroman-4-one	XPO
47	18.1	329.1043	(3*R*)-5,7-dihydroxy-6-methyl-8-methoxy-3-(4′-hydroxybenzyl)-chroman-4-one	XPO
50	19.29	313.1061	(3*R*)-5,7-dihydroxy-6,8-dimethyl-3-(4′-hydroxybenzyl)-chroman-4-one	XPO
51	19.35	329.1071	Isomer of (3*R*)-5,7-dihydroxy-6-methyl-8-methoxy-3-(4′-hydroxybenzyl)-chroman-4-one	GPO
56	20.69	299.0875	Isomer of (3*R*)-5,7-dihydroxy-6-methyl-3-(4′-hydroxybenzyl)-chroman-4-one	GPO
57	21.28	753.4075	3*β*-hydroxy-25*S*-spiriost-3-*O*-*β*-D-glucopyranosyl (1→4)-*β*-D-galactopyranoside	GPO
59	23.46	313.1019	Isomer of (3*R*)-5,7-dihydroxy-6,8-dimethyl-3-(4′-hydroxybenzyl)-chroman-4-one	GPO

Peak numbers were consistent with Supplementary Table S1.

### 3.3 Quantitative analysis of the PO samples by UHPLC-CAD with two commercial specifications

The furostonal saponin polygonatumoside F showed the higher content in all PO samples and thus could serve as the chemical marker for quality control, while the other components polygodoraside G and timosaponin H1 were screened out as chemical markers in XPO and GPO. To further understand the variation in the contents of the main steroidal saponins, a UHPLC-CAD approach was developed for the quantitative analysis of three furostanol saponins (polygodoraside G, polygonatumoside F, and timosaponin H1) in 27 samples with two commercial specifications; the chemical structures showed in Supplementary Figure S3.

The performance of Waters ACQUITY BEH C18 and ACQUITY CORTECS T3 was compared. Polygodoraside G and polygonatumoside F could be separated by Waters ACQUITY™ HSS T3. Therefore, the ACQUITY™ HSS T3 column was used for further separation. The column temperature and injection volume were also considered, and finally, the gradient was optimized under the condition of isocratic elution for the separation of the compounds. Subsequently, the established method was validated. The regression equations of the three analytes were calculated in the form of *Y* = a*X* ^2^ + b*X* + c; *X* and *Y* indicated the concentrations of the compound and the corresponding peak area, respectively. A good linear correlation of the three analytes was gained (*r* ^2^ > 0.999) with a specific concentration range. Due to the high sensitivity of CAD, the LOD and LOQ were in the nanogram level. The results of the method validation are summarized in Table 2. The data indicated that the UHPLC-CAD method possessed good accuracy with recoveries from 97.38% to 106.76%, and all the RSDs of precision, stability, and repeatability were <3%.

**TABLE 2:** Calibration curves, linear range, LOD, LOQ, recovery, precision, repeatability, and stability of three investigated analytes in PO.

No	Analyte	Calibration equation	*r* ^2^	Range (µg/mL)	LOD (ng)	LOQ (ng)	Recovery (%)	Precision (%)	Stability (%)	Repeatability (%)
1	Polygodoraside G	*Y* = −104.67*X* ^*2*^+30.31*X*	0.9999	0.23–30.00	0.04	2.00	105.10	0.83	1.83	2.75
2	Polygonatumoside F	*Y* = −76.39*X* ^*2*^+32.44*X*	0.9998	0.47–60.00	0.12	2.00	97.38	1.05	1.05	2.98
3	Timosaponin H1	*Y* = −9.55*X* ^*2*^+27.68*X*	0.9999	0.23–30.00	0.10	4.00	106.76	0.86	2.70	2.13

**FIGURE 6:**
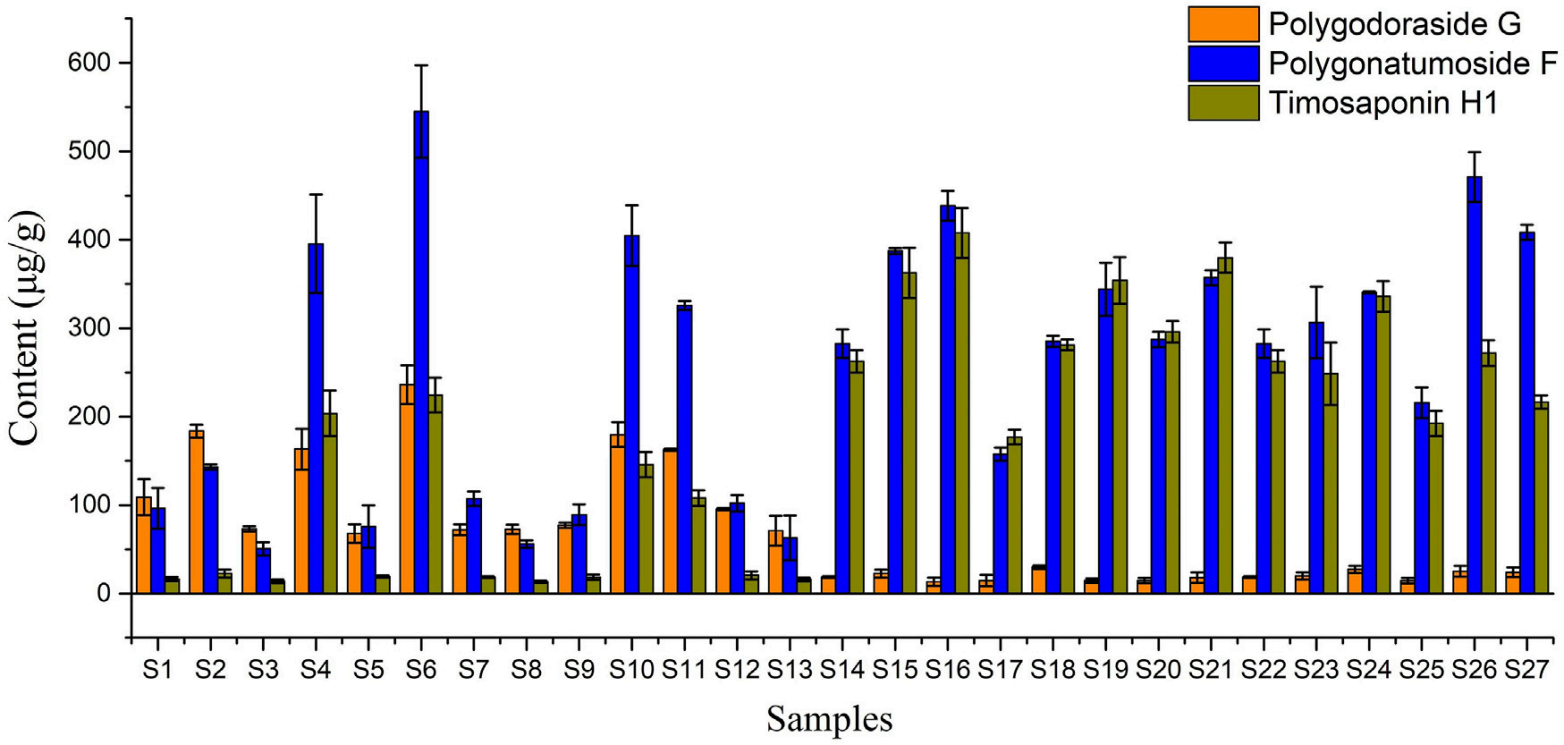
Contents of the three compounds in 27 batches of PO.

**FIGURE 7:**
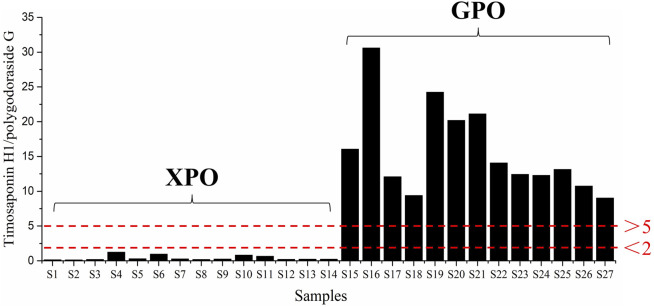
Ratio of timosaponin H1/polygodoraside G in 27 batches of PO.

Then, the developed UHPLC-CAD method was applied to determine the three targeted compounds in different batches of PO samples (Supplementary Figure S4). Each sample was determined in triplicate, and the contents of targeted analytes are presented in Table 3. The contents of polygodoraside G, polygonatumoside F, and timosaponin H1 ranged from 13.33 to 236.24 μg/g, 50.55–545.04 μg/g, and 13.34–407.83 μg/g, respectively. Considering the total content of the three compounds (Figure 6), the quality of GPO was stable in the range of 349.19–859.64 μg/g, while the quality of different batches of XPO varied greatly, and the total content was 137.70–1005.55 μg/g. Moreover, polygonatumoside F was the main characteristic compound in the samples of PO. The average content of polygonatumoside F in XPO was 195.49 μg/g and was lower in GPO with 329.35 μg/g, indicating that GPO should be focused upon from the perspective of saponin content. In addition, polygodoraside G was present in XPO with an average content of 113.04 μg/g, and that in GPO was 19.82 μg/g, while timosaponin H1 was mainly present in XPO with an average content of 78.85 μg/g and that in GPO was 291.22 μg/g; the content ratio of the two compounds in different specifications differed markedly. Therefore, it can be concluded that the samples with a ratio <2 of timosaponin H1/polygodoraside G were considered as XPO, and >5 were considered as GPO (Figure 7). In conclusion, the absolute content of the main characteristic components in PO has been investigated. We found that XPO and GPO have significant differences in the quality of medicinal materials. Thus, timosaponin H1/polygodoraside G seems to be a promising indicator to distinguish the two commercial specifications.

**TABLE 3:** Contents of three compounds in 27 batches of PO and the ratio of timosaponin H1/polygodoraside G.

Sample No.	Polygodoraside G (μg/g)	Polygonatumoside F (μg/g)	Timosaponin H1 (μg/g)	Total contents (μg/g)	Timosaponin H1/polygodoraside G
S1	109.03	96.39	16.62	222.04	0.15
S2	183.84	143.21	22.65	349.69	0.12
S3	73.18	50.55	13.97	137.70	0.19
S4	163.35	395.47	203.70	762.53	1.25
S5	67.80	75.76	19.28	162.83	0.28
S6	236.24	545.04	224.27	1005.55	0.95
S7	71.93	107.22	18.65	197.80	0.26
S8	72.69	56.02	13.34	142.06	0.18
S9	77.25	89.23	18.65	185.12	0.24
S10	179.74	404.75	145.79	730.27	0.81
S11	162.55	325.77	107.93	596.24	0.66
S12	95.22	93.21	19.31	218.00	0.20
S13	95.33	102.00	20.67	150.04	0.22
S14	71.04	63.06	15.94	563.50	0.22
S15	22.60	387.31	362.65	772.57	16.05
S16	13.33	438.48	407.83	859.64	30.60
S17	14.64	157.63	176.92	349.19	12.08
S18	29.98	285.11	281.11	596.19	9.38
S19	14.61	344.13	354.10	712.85	24.23
S20	14.66	287.17	295.84	597.67	20.18
S21	17.98	357.04	379.69	754.71	21.12
S22	18.65	282.42	262.43	563.50	14.07
S23	19.99	306.54	248.56	575.10	12.43
S24	27.33	340.59	335.93	703.85	12.29
S25	14.65	215.78	192.47	422.89	13.14
S26	25.31	470.98	271.79	768.08	10.74
S27	23.98	408.34	216.50	648.82	9.03

## 4 Conclusion

In order to understand the chemical composition of PO comprehensively, an effective and sensitive UHPLC-Q-TOF/MS method was developed for the characterization of PO with two commercial specifications. A total of 62 components were identified, of which 13 robust known chemical markers (including 6 flavonoids and 7 steroidal saponins) were screened out to differentiate Xiangyuzhu (XPO) and Guanyuzhu (GPO). A simultaneous determination method for polygodoraside G, polygonatumoside F, and timosaponin H1 was established and validated by UHPLC-CAD before applying it to determine the differences in the contents of the three components in XPO and GPO samples. The ratio of timosaponin H1/polygodoraside G can be used as an indicator to distinguish the two commercial specifications of PO*.* The samples with a ratio <2 are considered XPO and >5 are considered GPO.

In this study, the qualitative analysis and quantitative analysis of PO were carried out to elucidate the composition. The findings provided a method for distinguishing the two commercial specifications of PO in the market and laid a theoretical foundation for the appropriate utilization of the resources.

## Data Availability

The original contributions presented in the study are included in the article/Supplementary Material, further inquiries can be directed to the corresponding author.
