# Supplementary material for: Qualitative and quantitative studies on two commercial specifications of Polygonatum odoratum
Source: Front Chem. 2023 Feb 23;11:1146153. doi: 10.3389/fchem.2023.1146153 (PMC9995655; doi:10.3389/fchem.2023.1146153)
Supplement: Supplementary file 1 [file DataSheet1.docx]

Table S1 Characterization of chemical constituents in the crude extract of PO by UHPLC-Q-TOF/MS.

| Peak  No. | *t*_R_ (min) | Formula | [M-H]^-^ | | Error  (mDa) | Fragment ions | Identification | Source | |
| --- | --- | --- | --- | --- | --- | --- | --- | --- | --- |
|  |  |  | Meas. Mass | Calc. Mass |  |  |  | X | G |
| 1 | 3.9 | C_17_H_17_NO_4_ | 298.1056 | 298.108 | -2.4 | 280.0988, 179.0510, 145.0294 | *N*-*cis-p*-coumaroyloctopamine | + | + |
| 2 | 4.39 | C_18_H_19_NO_5_ | 328.1145 | 328.1185 | -4.0 | 310.1115, 161.0241, 132.0180 | *N*-*cis*-feruloyloctopamine (Zeng et al., 2020) | + | + |
| ^*^3 | 4.65 | C_17_H_17_NO_4_ | 298.1071 | 298.108 | -0.9 | 280.094, 160.0376, 145.0278, 119.0468, 117.0317 | *N*-*trans-p*-coumaroyloctopamine (Pang et al., 2020) | + | + |
| ^#^4 | 5.12 | C_54_H_96_O_33_ | 1271.5759 | 1271.5756 | 0.3 | 1109.5161, 1073.4901, 947.4603, 911.4377, 785.3995, 749.3846 | Kingianoside E+Glc+OH | + | - |
| ^*^5 | 5.34 | C_18_H_19_NO_5_ | 328.1163 | 328.1185 | -2.2 | 310.1057, 161.0224, 133.0461 | *N*-*trans-*feruloyloctopamine (Pang et al., 2020) | + | + |
| ^#^6 | 5.43 | C_57_H_94_O_29_ | 1241.5737 | 1241.5803 | -6.6 | 1205.5425, 1109.5120, 1079.5015, 947.4532, 911.4369, | Kingianoside E+Xyl+OH | + | - |
| ^#^7 | 6.52 | C_57_H_92_O_30_ | 1255.5535 | 1255.5595 | -6 | 1093.4962, 931.4557, 769.3910, 751.3771 | Polygodoraside F-Xyl+Glc | + | - |
| 8 | 6.71 | C_17_H_17_NO_3_ | 282.1079 | 282.113 | -5.1 | 273.1639, 243.1209 | *N*-*cis-p-*coumaroyltyramine (Ren et al., 2020) | + | + |
| ^*^9 | 6.85 | C_56_H_90_O_29_ | 1225.5461 | 1225.549 | -2.9 | 1093.5017, 1063.4921, 931.4497, 769.3924, 751.3911 | Polygodoraside F (Pang et al., 2020) | + | - |
| ^*^10 | 6.97 | C_56_H_90_O_29_ | 1225.5479 | 1225.549 | -1.1 | 1093.5293, 1063.5087, 931.4622, 769.4100, 751.4044 | Polygodoraside D (Pang et al., 2020) | + | - |
| 11 | 7.16 | C_45_H_74_O_20_ | 933.4664 | 933.4695 | -3.1 | 771.4195 609.3679, 591.3572, 429.2904 | Isomer of (25*R*,22*ξ*)-hydroxylwattinoside C | - | + |
| 12 | 7.44 | C_18_H_19_NO_4_ | 312.1165 | 312.1236 | -7.1 | 135.0423, 147.0401 | *N*-*cis*-feruloyltyramine (Ren et al., 2020) | + | + |
| ^#^13 | 7.65 | C_39_H_64_O_15_ | 771.4197 | 771.4169 | 2.8 | 609.3630, 463.2521, 447.3109 | (25*R*,22*ξ*)-hydroxylwattinoside C-Glc | - | + |
| ^#^14 | 7.7 | C_54_H_94_O_32_ | 1253.5642 | 1253.565 | -0.8 | 1091.5040, 929.4490, 767.3957, 749.3743 | Kingianoside H+3Glc | + | - |
| 15 | 7.79 | C_45_H_74_O_19_ | 917.4739 | 917.4746 | -0.7 | 771.4188, 609.3641, 463.2540, 447.3123 | Isomer of (3*β*,25*R*)-26-(*β*-D-glucopyranosyloxy)-  hydroxyfurost-5-en-3-yl 4-O-*β*-D-glucopyranosyl-*β*-D-  galactopyranoside | - | + |
| 16 | 7.95 | C_17_H_17_NO_3_ | 282.1137 | 282.113 | 0.7 | 273.1637, 243.1208 | N-*trans-p*coumaroyltyramine (Ren et al., 2020) | + | + |
| ^*^17 | 8.04 | C_57_H_94_O_30_ | 1257.5757 | 1257.5752 | 0.5 | 1095.5294, 933.4738, 771.4175 | Polygodoraside G (Pang et al., 2020) | + | + |
| 18 | 8.09 | C_44_H_72_O_18_ | 887.4561 | 887.464 | -7.9 | 741.4034, 609.3620, 579.3508,  447.3080 | Isomer of polygonoide B | - | + |
| 19 | 8.14 | C_51_H_84_O_25_ | 1257.5741 | 1257.5752 | -1.1 | 1095.5356, 933.4686, 771.4218 | (25*R*)-Polygodoraside G | + | - |
| ^*^20 | 8.16 | C_56_H_90_O_29_ | 1225.5444 | 1225.549 | -4.6 | 1093.5201, 1063.5038, 917.4827, 771.4178 | Polygodoraside A (Pang et al., 2020) | - | + |
| ^*^21 | 8.41 | C_56_H_92_O_29_ | 1227.563 | 1227.5646 | -1.6 | 1095.5287, 1065.5164, 933.4706, 771.4127 | Polygonatumoside F (Pang et al., 2020) | + | + |
| 22 | 8.5 | C_56_H_92_O_29_ | 1227.5575 | 1227.5646 | -7.1 | 1095.5320, 1065.5160, 933.4669, 771.4136 | (25*R*)-Polygonatumoside F | + | + |
| 23 | 8.64 | C_18_H_19_NO_4_ | 312.1199 | 312.1236 | -3.7 | 135.0423, 147.0401 | *N*-*trans*-feruloyltyramine | + | + |
| 24 | 8.7 | C_45_H_74_O_20_ | 933.4652 | 933.4695 | -4.3 | 771.4164, 609.3659, 591.3494, 567.2715, 465.2653 | Isomer of (25*R*,22*ξ*)-hydroxylwattinoside C | - | + |
| 25 | 9.26 | C_56_H_92_O_28_ | 1211.5618 | 1211.5697 | -7.9 | 1079.5381, 1049.5280, 917.4763, 755.4230 | Isomer of Timosaponin H1 | - | + |
| 26 | 9.63 | C_45_H_74_O_19_ | 917.4717 | 917.4746 | -2.9 | 755.4227, 591.3500 | (3*β*,25*R*)-26-(*β*-D-glucopyranosyloxy)-22-  hydroxyfurost-5-en-3-yl 4-O-*β*-D-glucopyranosyl-*β*-D-  galactopyranoside (Ren et al., 2020) | - | + |
| 27 | 10.03 | C_33_H_54_O_10_ | 609.3652 | 609.3639 | 1.3 | 447.3158 | Polygonatumoside G (Liu et al., 2018) | - | + |
| 28 | 10.2 | C_39_H_66_O_13_ | 741.4414 | 741.4425 | -1.1 | 579.3983, 433.3357 | (22*S*)-cholest-5-ene-1*β*,3*β*,16*β*,22-tetrol-1-O-*α*-*L*-rhamnopyranosyl-16-O-*β*-D-glucopyranoside (Quan et al., 2015) | + | - |
| ^*^29 | 10.38 | C_57_H_94_O_29_ | 1241.5813 | 1241.5803 | 1 | 1079.5420, 917.4802, 755.4265 | Polygodoraside H (Pang et al., 2020) | + | - |
| ^*^30 | 10.5 | C_50_H_80_O_24_ | 1063.4971 | 1063.4961 | 1 | 931.4229, 901,4410, 769.3884 | Polygodoraside B (Pang et al., 2020) | - | + |
| 31 | 10.56 | C_51_H_84_O_24_ | 1079.5264 | 1079.5275 | -1.1 | 917.4793, 755.4248 | ^*^(25*S*)-26-O-(*β*-D-glucopyranosyl)-furost-5-en3*β*,22*α*,26-triol 3-O-*β*-D-glucopyranosyl-(1→2)-*β*-D-glucopyranosyl-(1→4)-*β*-D-glucopyranoside (Pang et al., 2020) | - | + |
| 32 | 10.63 | C_16_H_14_O_6_ | 301.0716 | 301.0712 | 0.4 | 191.0311, 179.0370, 125.0212 | Disporopsin (Quan et al., 2015) | + | - |
| 33 | 10.79 | C_56_H_92_O_28_ | 1211.5667 | 1211.5697 | -3 | 1079.5300, 1049.5077, 917.4588, 755.4054 | ^*^Timosaponin H1 (Pang et al., 2020) | + | + |
| ^#^34 | 10.9 | C_47_H_76_O_21_ | 975.4761 | 975.4801 | -4 | 933.4755, 915.4627, 771.4144, 753.4074 | Acetyl-(25*S*,22*ξ*)-hydroxylwattinoside C | - | + |
| ^#^35 | 11.14 | C_57_H_92_O_29_ | 1239.5619 | 1239.5646 | -2.7 | 1077.5377, 915.4727, 753.4117 | Kingianoside D+2Glc | + | - |
| ^#^36 | 11.14 | C_55_H_98_O_31_ | 1253.595 | 1253.6014 | -6.4 | 1211.5792, 1079.5385, 1049.5275, 917.4746 | Acetyl-Timosaponin H1 | - | + |
| 37 | 11.21 | C_45_H_74_O_19_ | 917.4779 | 917.4746 | 3.3 | 755.4543, 593.3752, 575.3610 | (3*β*,25*R*)-26-(*β*-D-glucopyranosyloxy)-22-hydroxyfurost-5-en-3-yl 4-O-*β*-D-glucopyranosyl-*β*-D-galactopyranoside (Ren et al., 2020) | - | + |
| ^*^38 | 11.48 | C_56_H_90_O_28_ | 1209.552 | 1209.5541 | -2.1 | 1077.5208, 1047.5129, 915.4697, 753.4073 | Polygodoraside E | + | + |
| ^#^39 | 11.76 | C_47_H_76_O_20_ | 959.4821 | 959.4852 | -3.1 | 917.4806, 899.4655, 755.4191, 737.4115 | Acetyl-(3*β*,25*S*)-26-(*β*-D-glucopyranosyloxy)-22-hydroxyfurost-5-en-3-yl 4-O-*β*-D-glucopyranosyl-*β*-D-  galactopyranoside | - | + |
| ^*^40 | 12.82 | C_33_H_54_O_10_ | 609.3631 | 609.3639 | -0.8 | 447.3091 | Polygonatumoside G (Pang et al., 2020) | + | + |
| ^#^41 | 14.95 | C_51_H_80_O_25_ | 1091.49 | 1091.491 | -1 | 929.4390, 767.3913, 605.3387 | Kingianoside G+Glc | + | - |
| 42 | 15.2 | C_51_H_82_O_23_ | 1061.5146 | 1061.5169 | -2.3 | 929.4390, 899.4225, 767.3950 | Isomer of 3-*O-β*-D-glucopyranosyl-(1→2)-[*β*-D- glucopyranosyl-(1→3)]-*β*-D-glucopyranosyl (1→4)-*β*-D-galacopyranosyl-diosgenin | + | - |
| ^*^43 | 15.47 | C_56_H_90_O_27_ | 1193.5543 | 1193.5591 | -4.8 | 1061.5265, 1031.5209, 899.4713 | Typaspidoside L (Pang et al., 2020) | - | + |
| 44 | 15.73 | C_18_H_18_O_7_ | 345.0939 | 345.0974 | -3.5 | 330.0732, 237.0928, 155.0305 | 5,7-dihydroxy-6-methoxyl-8-methyl-3-(2′,4′-dihydroxybenzyl) chroman-4-one | - | + |
| 45 | 16.12 | C_17_H_16_O_5_ | 299.0919 | 299.0919 | 0 | 193.0478 | Isomer of (3R)-5,7-dihydroxy-6-methyl-3-(4′-hydroxybenzyl)-chroman-4-one | + | + |
| 46 | 17.47 | C_17_H_16_O_5_ | 299.0952 | 299.0919 | 3.3 | 282.9475, 193.0473 | ^*^(3R)-5,7-dihydroxy-6-methyl-3-(4′-hydroxybenzyl)-  chroman-4-one (Pang et al., 2020) | + | - |
| ^*^47 | 18.08 | C_18_H_18_O_8_ | 329.1043 | 329.1025 | 1.8 | 223.0574, 208.0370 | (3R)-5,7-dihydroxy-6-methyl-8-methoxy-3-(4’-hydroxybenzyl)-chroman-4-one (Pang et al., 2020) | + | + |
| 48 | 18.75 | C_50_H_78_O_23_ | 1045.4905 | 1045.4856 | 4.9 | 1045.4797, 913.4522, 883.4460, 751.3910, 589.3411 | (25*S*)-spirostan-5-en-12-one-3-O-D-glucopyranosyl- (1→2)-O-[*β*-D-xylopyranosyl(1→3)]-O-*β*-D-glucopyranosyl (1→4)-*β*-D-galactopyranoside (Ren et al., 2020) | + | + |
| 49 | 18.95 | C_51_H_80_O_24_ | 1075.4891 | 1075.4961 | -7 | 913.4512, 751.4008, 589.3360 | Isomer of cyrtonemoside A | + | - |
| ^*^50 | 19.29 | C_18_H_18_O_5_ | 313.1061 | 313.1076 | -1.5 | 207.0653, 179.0683 | (3R)-5,7-dihydroxy-6,8-dimethyl-3-(4′-hydroxyben-zyl)-chroman-4-one (Pang et al., 2020) | + | - |
| 51 | 19.34 | C_18_H_18_O_8_ | 329.1071 | 329.1025 | 4.6 | 193.0468, 139.0356 | Isomer of (3*R*)-5,7-dihydroxy-6-methyl-8-methoxy-3-  (4’-hydroxybenzyl)-chroman-4-one | - | + |
| 52 | 19.54 | C_50_H_80_O_23_ | 1047.4937 | 1047.5012 | -7.5 | 915.4636, 885.4626, 753.4102, 591.3561 | (25*S*)-(3*β*,14*α*)-dihydroxy-spirost-5-en-3-*O-β*-D-glucopyranosyl-(1→2)-[*β*-D-xylopyranosyl-(1→3)]-*β*-D-glucopyranosyl (1→4)-*β*-D-galacopyranoside (Pang et al., 2020) | + | + |
| 53 | 19.74 | C_50_H_80_O_23_ | 1047.5023 | 1047.5012 | 1.1 | 915.4617, 885.4522, 753.4077, 591.3516 | (25*R*)-(3*β*,14*α*)-dihydroxy-spirost-5-en-3-*O-β*-D-glucopyranosyl-(1→2)-[*β*-D-xylopyranosyl-(1→3)]-*β*-D-glucopyranosyl (1→4)-*β*-D-galacopyranoside (Ren et al., 2020) | - | + |
| 54 | 20.08 | C_18_H_18_O_8_ | 329.1008 | 329.1025 | -1.7 | 193.0116, 165.0177 | Isomer of (3*R*)-5,7-dihydroxy-6-methyl-8-methoxy-3-  (4’-hydroxybenzyl)-chroman-4-one | - | + |
| 55 | 20.4 | C_19_H_20_O_7_ | 359.1087 | 359.1131 | -4.4 | 169.0436 | Ophiopogonanone E (Ren et al., 2020) | + | - |
| 56 | 20.66 | C_17_H_16_O_5_ | 299.0875 | 299.0919 | -4.4 | 177.0158 | Isomer of (3*R*)-5,7-dihydroxy-6-methyl-3-(4′-hydroxy-benzyl)-chroman-4-one | - | + |
| 57 | 21.27 | C_39_H_62_O_14_ | 753.4075 | 753.4061 | 1.4 | 591.3508, 573.3646 | 3*β*-hydroxy-25*S*-spiriost-3-O-*β*-D-glucopyranosyl (1→4)-*β*-D-galactopyranoside (Ren et al., 2020) | - | + |
| 58 | 21.45 | C_39_H_62_O_14_ | 753.4061 | 753.4061 | 0 | 591.3512, 573.3589 | 3*β*-hydroxy-25*R*-spiriost-3-O-*β*-D-glucopyranosyl (1→4)-*β*-D-galactopyranoside (Ren et al., 2020) | - | + |
| 59 | 23.44 | C_18_H_18_O_5_ | 313.1019 | 313.1076 | -5.7 | 201.1103, 192.0375, 135.0056 | Isomer of (3*R*)-5,7-dihydroxy-6,8-dimethyl-3-(4′-  hydroxybenzyl)-chroman-4-one | - | + |
| 60 | 23.73 | C_51_H_82_O_23_ | 1061.5176 | 1061.5169 | 0.7 | 899.4650, 737.4129, 575.3652 | 1. *O-β*-D-glucopyranosyl-(1→2)-[*β*-D-glucopyranosyl-(1→3)]-*β*-D-glucopyranosyl (1→4)-*β*-D-galacopyranosyl-diosgenin (Zhao et al., 2019) | + | - |
| 61 | 24.16 | C_50_H_80_O_22_ | 1031.4999 | 1031.5063 | -6.4 | 899.4958, 737.4155 | 1. *O-β*-D-glucopyranosyl-(1→2)-[*β*-D-xylopyranosyl-(1→3)]-*β*-D-glucopyranosyl (1→4)-*β*-D-galacopyranosyl-diosgenin (Zhao et al., 2019) | + | + |
| 62 | 24.29 | C_50_H_80_O_22_ | 1031.5035 | 1031.5063 | -2.8 | 899.4688, 737.4174 | 1. *O-β*-D-glucopyranosyl-(1→2)-[*β*-D-xylopyranosyl-(1→3)]-*β*-D-glucopyranosyl (1→4)-*β*-D-galacopyranosyl-yamogenin (Liu et al., 2018) | + | + |

*Compared with reference component.

# Tentatively identified as novel compounds.


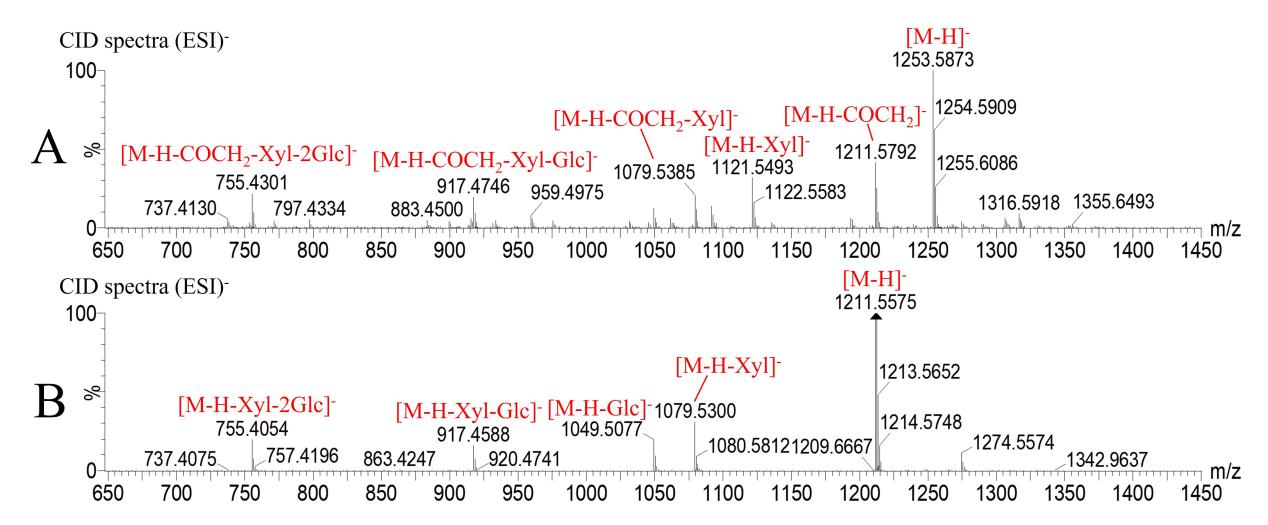


Figure S1. Mass spectrogram in negative mode of acetyl-timosaponin H1 and timosaponin H1. (A) CID spectra of acetyl-timosaponin H1. (B) CID spectra of timosaponin H1.


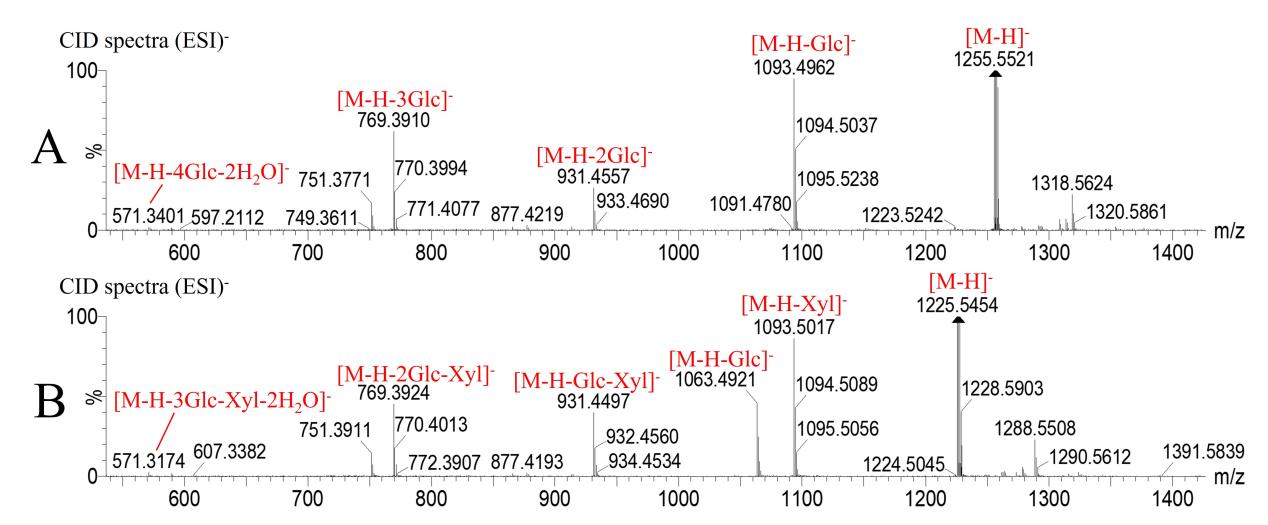
Figure S2. Mass spectrogram in negative mode of polygodoraside F-Xyl+Glc and polygodoraside F. (A) CID spectra of polygodoraside F-Xyl+Glc. (B) CID spectra of polygodoraside F.

Figure S3. Chemical structures of polygodoraside G, polygonatumoside F and timosaponin H1.


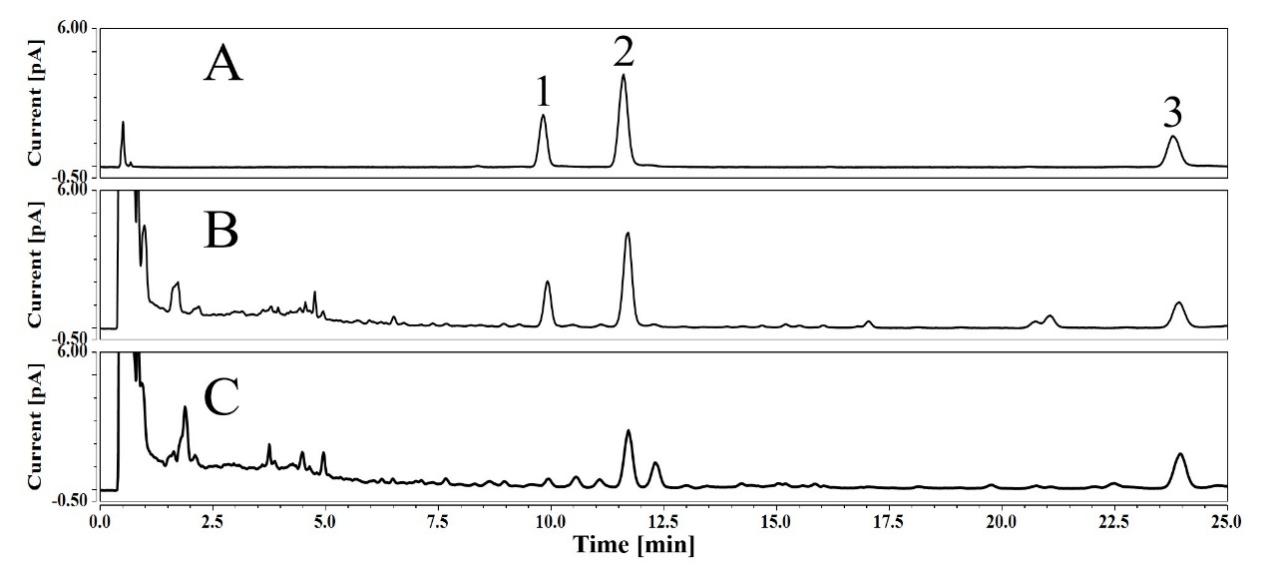


Figure S4. Chromatogram of reference components and PO samples analysed by UHPLC-CAD (A) chromatogram of reference components; (B) chromatogram of XPO sample; (C) chromatogram of GPO sample; 1, polygodoraside G; 2, polygonatumoside F; 3, timosaponin H1.
